# Current Literature for Veterinarians

**Published:** 1894-01

**Authors:** 


### EDITORIAL.

### CURRENT LITERATURE FOR VETERINARIANS.

The announcement of the establishment of a new veterinary periodical by the University of Pennsylvania, Veterinary Department, brings up recollections of our own experience in editing a journal upon a special subject. In musing over the demands of the veterinarian for the reading which he wants to aid him in his practice, to educate him for contact with the varied world with which he is thrown, and to distract himself with in his idle moments, we soon found ourselves deep in the files of our exchanges and current literature, and realized what an atom a single publication is in the mass of good, bad, and worse which is published. The growing circulation of the JOURNAL, not only among veterinarians, but also among physicians, scientists, and laymen interested in animals, is evidence that it has been appreciated, and has succeeded in supplying the news and information which is asked for. A veterinary publication cannot live as do newspapers, secular periodicals, and many medical journals, upon the advertisements which are taken with little scrutiny and mixed with reading matter to catch any or all classes of readers. The readers of a journal upon a special subject demand that it shall give its semi-indorsement of its advertisers only to those of reputation, so that non-professional supplies are only advertised in its pages when there is some reason for a special demand for them by the readers. A veterinary journal in its reading matter, in the same way, must supply its subscribers with all that there is of importance and value upon strictly professional subjects, and with such other matter as is allied to the general information and education of its clientele. With the progressive spirit of the veterinary community in America, and the present diversified opinions amongst the fraternity as to the manner in which greater unity of system of education is to be attained, it would be both arrogant and inexpedient to "EDIT" all the material which reaches us, as can be done upon a still more specialized subject, and it would be improper for the editorial pen to advocate too strongly many of the new theories in veterinary medicine, no matter how enthusiastically we may favor them. It is for us, only, to select to the best of our judgment original manuscripts and reproductions from other journals which seem best suited to the wants of our readers. Our limited four or five score of pages in a number force us to reject and omit much which we would like to publish. How well we have succeeded, we compliment ourselves upon, by the increase of our subscription list.

The following condensed review of our file of exchanges, we believe, will be of value in guiding those who wish to extend their reading beyond the pages of the JOURNAL, which we hope will continue to retain its old friends as it adds new ones in the coming year.

### VETERINARY JOURNALS.

### AMERICA.

THE JOURNAL OF COMPARATIVE MEDICINE AND VETERINARY ARCHIVES. Monthly, Vol. XV., No. 1, January, 1894. Editors, W. A. Conklin, Ph.D., D.V.S., and Rush Shippen Huidekoper, M.D., Veterinarian (Alfort), 155 West 56th Street, New York City.

The JOURNAL, which has now reached its fifteenth year, was established in New York, and continued as a quarterly publication for ten years, during which time Dr. Conklin was the guiding editor. Through his intimate affiliation with the scientific world most valuable papers upon subjects of original research in Comparative medicine were obtained. Four years ago Dr. Huidekoper joined the editorial staff, and the JOURNAL was transformed into a monthly periodical.

It offers to the veterinary profession, to physicians interested in comparative medicine, and to the intelligent agriculturist, stock breeder and horseman, a complete record of the discoveries in comparative medicine, and of the advance in veterinary matters. All matters of general interest in animal industry, the breeding, care of, shoeing, use of, and commerce in animals, are carefully reported. These allied subjects are as useful to the horseman and agriculturist as they are necessary to the veterinarian.

The JOURNAL publishes all important mattters pertaining to comparative medicine, clinical records, laboratory research, and animal industry. Illustrations are used whenever they can aid in rendering the writings more clear.

Full reports of the veterinary schools of the United States and Canada, and the proceedings of the local, State and United States Veterinary Medical Associations are published promptly.

The JOURNAL is the organ of no school, but is the representative of the veterinary profession of the United States and Canada.

AMERICAN VETERINARY REVIEW. Monthly. Vol. XVII. No. 9, December, 1893. Edited by A. Liautard, M.D., V.M., American Veterinary College, 141 West 54th Street, New York. The REVIEW was established and supported for a number of years by the United States Veterinary Medical Association, until it was placed upon a firm basis, when it was given to Prof. Liautard, by whom it has been conducted since that time. It publishes original and selected articles upon veterinary matters, with numerous translations and reports of current society proceedings.

NEW YORK VETERINARY JOURNAL AND RECORD OF COMPARATIVE MEDICINE. Quarterly. Vol. II, No. 2, November, 1893. Editors, Jacob Homer Gardner, Ph.D., D.V.S., and Theodore A. Keller, D.V.S., 207 West 42nd Street, New York. A small quarterly upon general veterinary matters.

VETERINARY MAGAZINE, Monthly. To appear January, 1894. University of Pennsylvania, Veterinary Department, Philadelphia, Penn. The prospectus, just received, announces:

"The 'Veterinary Magazine' will be edited by Simon J. J. Harger, V.M.D., Professor Veterinary Anatomy and Zootechnics; Leonard Pearson, B.S., V.M.D., Professor of Theory and Practice; Leo Breisacher, M D., V.M D., Professor of Physiology, and John William Adams, A.B., V.M.D., Assistant Professor of Surgery, Obstetrics and Farriery—all members of the Faculty of the Veterinary Department, of the University of Pennsylvania; and will have as collaborators a number of the most eminent veterinary practitioners and scientists of America and Europe."

ENGLAND AND SCOTLAND.

THE JOURNAL OF COMPARATIVE PATHOLOGY AND THERAPEUTICS. Quarterly. Edited by J. M'Fadyean, M.B., B. Sc., F. R. S. E., Royal Veterinary College, London. Vol. VI, Part 3, September, 1893.

"The Journal was founded in the beginning of 1888, with the object of stimulating scientific research and accurate clinical observation regarding the diseases of the lower animals. The present number will indicate the scope and character of the matter which it is intended to gather into the Journal

Stated briefly, the leading features of the publication are:

1. Original or translated Monographs and Essays by the most eminent authors.

2. Editorial articles on current topics in the domain of Medical Science.

3. Short Clinical Reports of interesting cases in veterinary practice.

4. Reviews and notices of important recent publications (British and Foreign) relating to the diseases of the lower animals.

5. Abstracts of the more important articles in French, German and Italian Veterinary Periodicals.

6. Illustrations freely introduced wherever they are required to elucidate the text."

THE VETERINARIAN. Monthly. Vol. LXVI. Edited by Professors Macqueen, Simonds, Brown, Axe, and McCall; London. *The Veterinarian* is the oldest English veterinary periodical, founded 1828. It publishes original articles upon veterinary subjects and full reports of the proceedings of the Royal College of Veterinary Surgeons, English and Scotch societies.

THE VETERINARY JOURNAL AND ANNALS OF COMPARATIVE PATHOLOGY. Monthly. Vol. XXXVII, 1893. Edited by George Fleming, C.B., LL.D., F.R.C.V.S.; London. Treats of the subjects of the same nature in general as *The Veterinarian.*

THE VETERINARY RECORD. Weekly. Vol. VI. commenced July, 1893. Edited by ——, London. A bright weekly with short articles of practical interest.

FRANCE.

RECUEIL DE MÉDECINE VÉTÉRINAIRE. Bi-monthly: Series VII, Vol. X, 1893. Edited by the Faculty of the Veterinary School at Alfort (Seine-France) with the concours of numerous French veterinarians. *The Recueil*, established in 1824, is the oldest and one of the most valuable of veterinary publications. Alternate numbers contain the proceedings of the Société Centrale de Médecine Vétérinaire.

REVUE VÉTÉRINAIRE (Ancien Journal des Vétérinaires du Midi). Monthly. 18th (50th) year. Editors, Faculty of the Veterinary School of Toulouse (France).

General veterinary articles.

JOURNAL DE MÉDECINE VÉTÉRINAIRE ET DE ZOOTECHINE. Monthly. 3d Series, Vol. XVIII; XLIV of the collection. Edited by the Faculty of the Veterinary School at Lyon (France).

General veterinary articles.

BELGIUM.

ANNALES DE MÉDECINE VÉTÉRINAIRE. Vol. XLII, 1893. Edited by the Faculty of the State Veterinary School at Cureghem, Brussels.

General veterinary articles.

ITALY.

GIORNALE DI VETERINARIA MILITARE, Rivista Mensile di Ippologia. Monthly. Vol. IV. Edited by Dott. L. Baruchello, Rome.

General veterinary articles and special practical matters concerning military application.

LA CLINICA VETERINARIA. Three numbers a month. Vol. XVI, 1893. Edited by Prof. Dr. Lanzillotti-Buonsanti, director of the Veterinary School, Milan.

General veterinary articles, scientific and practical translations from the German.

IL MODERNO ZOOIATRO. Bi-Monthly. Vov. IV, 1893. Edited by Roberto Bassi, Turin.

Veterinary and zootechnic subjects are treated, including the records of racing.

GIORNALE DE ANATOMIA, FISIOLOGIA E PATOLOGIA DEGLI ANIMALI. Vol. XXV, 1893. Edited by L. Lombardini, P. Oreste, A. Gilvesbuni, I. Vigezzi and others. Pisa.

Original articles, extracts and translations.

GERMANY.

ZEITSCHRIFT FUR VETERINARKUNDE, mit besonderer Berücksiehtigung der Hygiene. Organ für die Rossärzte der Armee. Vol. V, 1893. Monthly. Edited by Oberrossarzt G. Koenig, inspector of the Royal Military Veterinary School, Berlin, Germany.

Practical veterinary articles and horseshoeing.

REPETORIUM DER TIERHEILKUNDE, angefangen 1840, von O.-M.-R. Dr. V. Hering. Zeitschrift für tierärztliche Chirurgie. Edited by Prof. L. Hoffman, Royal Wurtemberg Veterinary High School, Stuttgart.

Rich in short reviews from other journals.

ARCHIV FUR WISSENSCHAFTLICHE U. PRAKTISCHE THIERHEILKUNDE. Six numbers a year. Vol. XIX, 1893. Edited by Prof. Dr. C. Dammann, Geh. Reg.- und Med.-Rath und Direktor der Königl. Thierärztl. Hochschule in Hannover; Prof. Dr. W.

Ellenberger, Med.-Rath und Lehrer an der Königl. Thierärztl. Hochschule in Dresden; Prof. C. E. Müller, Prof. Dr. J. W. Schütz, Lehrer an der Königl. Thierärztlichen Hochschule in Berlin, und Prof. Dr. O. Siedamgrotzky, Ober-Med.-Rath und Lehrer an der Königl. Thierärztlichen Hochschule in Dresden.

Berlin, Germany.

An advanced scientific veterinary publication.

Oesterreichische Monatschrift fur Theirheilkunde und Revue fur Thierheilkunde und Thierzucht. Vol. XVIII, 1893. Edited by Alois Koch, K. K. Bezirksthierarzt, Vienna (Austria).

A practical clinical journal frequently illustrated. Contains good reviews.

Monatschrift des Vereins des Thierarzte in Oesterreich. Monthly. Vol. XVI, 1893. Edited by the Society, Vienna.

Practical short articles and matters of local affairs.

Thiermedizinische Vortrage. Monthly. Vol. III, 1893. Edited by Dr. George Schneidemuhl, University of Riel.

Original articles and extensive reviews.

Thierarzliche Mittheilungen.

Wochenschrift fur Thierheilkunde und Viehzucht. Weekly, XXXVII year. Edited by M. Albrecht and Ph. J. Goring, Munich, Bavaria.

Short, practical articles.

Berliner Thierarztliche Wochenschrift. Edited by Dr. W. Dieckerhoff, Dr. R. Schmaltz, Professoren an der thierärztlichen Hochschule zu Berlin. Dr. R. Lothes, c. Departements- und Kreisthierarzt zu Koeln.

A practical clinical weekly.

SPAIN.

Gaceto Medico-Veterinaria. Rivista quincenal de medicina comparada, higiene, bacteriologia, agricultura, zootecnia, etc., é intereses profesionales. 2nd Series, Vol. XVII. Edited by Director D. Eusebio Molina Serrano, Madrid.

General veterinary and allied subjects.

SWITZERLAND.

Schweitzer Archiv fur Thierheilkunde, 6 numbers a year, vol. XXXV, 1893. Edited by Dr. A. Guillebean and Prof. E. Zschokke, Turich. General veterinary articles.

HOLLAND.

TIJDSCHRIFT VOOR VEEARTSENIJKUNDE EN VEETEELT. 6 numbers a year, Vol. XXI. Edited by D. F. van Esoeld, Dr. L. J. van der Harst and W. C. Schimmel, Royal Veterinary School, Utrecht.

General veterinary articles.

JAVA.

VEEARTSENIJKUNDIGE BLADEN VOOR NEDERLANSCH-INDIE. Vol. VII, 1893. Edited by the Veterinary Society of the Dutch Indies, Batavia, Java.

A live, active, scientific and practical journal.

RUSSIA.

ARCHIVES OF VETERINARY SCIENCE. Quarterly. Vol. VI, 1893. Edited under the auspices of the Government Medical Department, St. Petersburg. General veterinary matters and numerous translations,

DENMARK.

TIDSSKRIFT FOR VETERINÆRER. Monthly. Edited by Prof. Dr. H. Krabbe, Copenhagen. General Veterinary articles.

SWEDEN.

TIDSSKRIFT FOR VETERINAR-MEDICIN OCH HUSDJURSSKOTSEL. Vol. XII, 1893. Edited by C. A. Lindquist, Professsor of the Royal Veterinary Institution, Stockholm. Scientific and practical veterinary articles.

INDIA.

INDIAN VETERINARY JOURNAL. Edited by Dr. Henry T. Pease, Lahore. Printed in Hindoostanee.

To be continued by a review upon journals publishing matters concerning subjects allied to veterinary affairs.

